# Experimental and density functional theory studies on some metal oxides and the derived nanoclusters: a comparative effects on human ferritin

**DOI:** 10.1186/s11671-023-03922-5

**Published:** 2024-01-15

**Authors:** Zahraa S. Al-Garawi, Ahmad H. Ismail, Duaa H. Hillo, Füreya Elif Öztürkkan, Hacali Necefoğlu, Gehad G. Mohamed, Abanoub Mosaad Abdallah

**Affiliations:** 1 Department of Chemistry, College of Sciences, Mustansiriyah University, Baghdad, 10001 Iraq; 2 Department of Chemical Engineering, Kafkas University, 36100 Kars, Turkey; 3 Department of Chemistry, Kafkas University, 36100 Kars, Turkey; 4 International Scientific Research Centre, Baku State University, 1148 Baku, Azerbaijan; 5 Chemistry Department, Faculty of Science, Cairo University, Giza, 12613 Egypt; 6 Nanoscience Department, Basic and Applied Sciences Institute, Egypt-Japan University of Science and Technology, New Borg El Arab, Alexandria 21934 Egypt; 7 Narcotic Research Department, National Center for Social and Criminological Research (NCSCR), Giza, 11561 Egypt

**Keywords:** Green synthesis, Ginger plant extract, Metal oxides nanoparticles, DFT calculations, Serum ferritin levels, Molecular docking simulations

## Abstract

A comprehensive investigation into the green synthesis of metal oxide nanoparticles (NPs) has garnered significant attention due to its commendable reliability, sustainability, and environmentally friendly attributes. Green synthesis methods play a crucial role in mitigating the adverse effects associated with conventional approaches employed for nanostructure preparation. This research endeavors to examine the impact of ginger plant extract-assisted green synthesis of metal oxides NPs on the serum ferritin levels of anemic diabetic patients in vitro, focusing specifically on α-Fe_2_O_3_ and ZnO NPs. Sixty diabetic volunteers with anemia (35–50 years) and thirty healthy volunteers were enrolled as controls. The assessment was conducted using the VIDAS Ferritin (FER) assay. Photoluminescence (PL) spectroscopy measurements were performed to elucidate the intrinsic and extrinsic transitions of these NPs, affirming the successful formation of α-structured iron oxide. Density functional theory (DFT) calculations were carried out at the B3LYP/6-311++G(d,2p) level of theory to investigate the geometry optimization and molecular electrostatic potential maps of the NPs. Furthermore, TD-DFT calculations were employed to explore their frontier molecular orbitals and various quantum chemical parameters. The binding affinity and interaction types of ZnO and α-Fe_2_O_3_ NPs to the active site of the human H-Chain Ferritin (PDB ID: 2FHA) target were determined with the help of molecular docking. Results unveiled the crystalline structure of ZnO and the α-structure of α-Fe_2_O_3_. Analysis of the frontier molecular orbitals and dipole moment values demonstrated that ZnO (total dipole moment (D) = 5.80 µ) exhibited superior chemical reactivity, biological activity, and stronger molecular interactions with diverse force fields compared to α-Fe_2_O_3_ (D = 2.65 µ). Molecular docking of the metal oxides NPs with human H-chain ferritin provided evidence of robust hydrogen bond interactions and metal-acceptor bonds between the metal oxides and the target protein. This finding could have a great impact on using metal oxides NPs-ferritin as a therapeutic protein, however, further studies on their toxicity are required.

**Graphical abstract:**

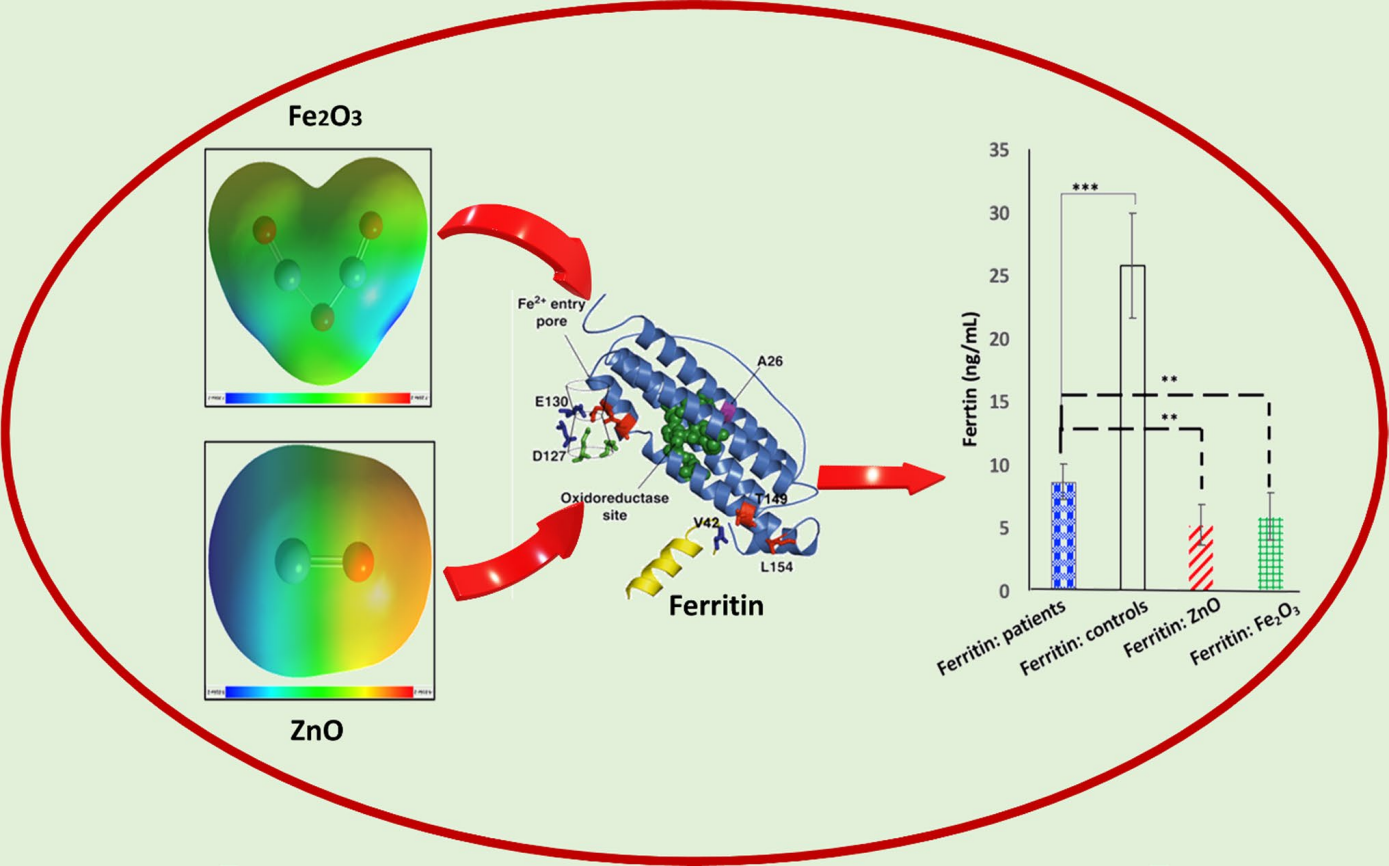

## Introduction

Metal oxide nanoparticles (MO NPs) are gaining increasing value in various industries due to their superior chemical, physical, and electrical properties compared to their bulk counterparts. These adaptable materials find applications in diverse sectors, including personal care, medical technology, energy, water treatment, and environmental cleanup [1, 2]. Nanoparticles exhibit unique characteristics arising from their distinct sizes, shapes, morphologies, compositions, agglomerations, and regularity states [3–6]. They possess a surface-to-volume ratio 35–45% higher than that of atoms or larger particles, resulting in a large specific surface are, which contributing to multifunctionality of those nanoparticles. In the medical field, MO NPs have demonstrated numerous applications, such as antibacterial properties, drug delivery systems, cancer therapy, antioxidant effects, and antidiabetic properties [7–15]. Methods for nanoparticle synthesis included thermal breakdown, sputtering, mechanical milling, laser ablation, and nanolithography. The potential and utility of nanoparticles are determined by their unique properties. However, their practical implementation is hindered by the use of potentially harmful and toxic materials, costly investments, toxic environments, high energy requirements, slow reaction times, and environmentally unfriendly byproducts [16].

An environmentally friendly method known as “biosynthesis” involved the formation of metal atom clusters that eventually developed into nanoparticles. Biosynthesis replaces expensive and toxic chemicals with plant extracts [17–21]. Recently, biological approaches utilizing natural resources such as plants, bacteria, fungi, seaweed, polysaccharides, biodegradable polymers, plant-derived materials, and algae, combined with environmentally friendly green chemistry-based techniques, have replaced traditional methods for metal and MO NPs synthesis. Biosynthetic approaches have garnered significant interest due to their environmental friendliness, simplicity, economic viability, and clean technology. These methods eliminate the need for hazardous chemicals and do not produce impurities or byproducts. Plant extracts have received particular attention among the various bioentities, as they possess unique natural properties that enable the reduction and stabilization of MO NPs in a single synthesis stage. Natural organic phytoconstituent biomolecules found in plant extracts, such as alkaloids, flavonoids, saponins, steroids, terpenoids, and tannins, operate as reducing and stabilizing agents due to their diverse and complex compositions [22–27].

DFT calculations, a type of quantum–mechanical (QM) simulation, are widely employed to investigate the electronic structure of chemical systems. DFT simulations have undergone extensive development and offer a high degree of precision in predicting molecular structures, geometries, and reactivity of chemical compounds [28–31]. By leveraging the fundamental principles of quantum mechanics, these calculations provide quantitative analysis of material properties. Molecular docking, a well-known in silico structure-based technique, plays a crucial role in drug development. It enables the identification of compounds with therapeutic potential without prior knowledge of the target modulators' chemical composition [32]. Molecular docking facilitates the prediction of interactions between ligands and targets at the molecular level, allowing for the characterization of structure–activity correlations (SAC) [33]. The ability to forecast binding affinity and evaluate interactive modes has become essential in computer-assisted drug design [34–36]. By testing compounds computationally, fewer compounds need to undergo empirical screening, resulting in time and cost savings compared to traditional physical studies [37].

In our previous study, we successfully synthesized ZnO NPs and α-Fe_2_O_3_ NPs using a green method that involved the utilization of ginger plant extract [38, 39]. The biodiversity of plant enables numbers of phytochemicals or also called secondary metabolites, especially in leaves, such as phenols, flavonoids, terpenoids, amides, aldehyde and ketones. The advantage of these secondary metabolites is to act as reducing agent which reduce metal precursor into metal nanoparticle. The reaction parameters like solvent and precursor concentration, reaction time, temperature, pressure and pH showed effect on synthesis of metal nanoparticles by green method [5, 6].

The synthesized NPs were characterized using various techniques, including ultraviolet–visible spectroscopy (UV–VIS), field emission scanning electron microscopy (FE-SEM), X-ray diffraction (XRD), and transmission electron microscopy (TEM). In this present work, we aimed to assess the impact of these MO NPs on the serum ferritin levels of anemic diabetic patients. We employed the VIDAS Ferritin (FER) assay, which provided quantitative measurements of serum ferritin levels. Furthermore, we utilized photoluminescence (PL) spectroscopy to gain insights into the intrinsic and extrinsic transitions of these MO NPS. We also conducted density functional theory DFT calculations to explore the geometry optimization and molecular electrostatic potential maps of the synthesized iron oxide and zinc oxide NPs. Moreover, time-dependent-density functional theory (TD-DFT) calculations were employed to investigate their frontier molecular orbitals and various quantum chemical parameters. To evaluate the interaction between the MO NPs and the target protein, human H-chain ferritin, we performed molecular docking simulations. This in silico approach allowed us to assess the binding affinity and potential molecular interactions between the nanoparticles and the ferritin protein.

Through this comprehensive study combining experimental and computational techniques, we aimed to deepen our understanding about effects of iron oxide and zinc oxide NPs on serum ferritin levels and their interaction with the human H-chain ferritin protein. These findings contribute to the explore the potential applications of these MO NPs—ferritin as therapeutic protein of anemic diabetic patients.

## Experimental

### Ginger extract and metal oxide NPsc [38, 39]

Ginger extract, ZnO NPs and α-Fe_2_O_3_ NPs were prepared and characterized in our pervious published works [38, 39]. Briefly, the process involved washing the dried ginger plant with deionized water, followed by grinding it into a fine powder. A weight of 10 g of the powdered plant was then dissolved in 200 mL of deionized water and boiled for 15 min, resulting in a yellow-colored solution. After cooling and filtering the solution, the yellow filtrate was used as a reducing and stabilizing agent for the synthesis of metal oxide nanoparticles from the ginger extract.

To prepare ZnO NPs, 2.07 g of Zn(NO_3_)_2_·6H_2_O were dissolved in 150 mL of deionized water to achieve a final concentration of 0.1 M. Next, 20 mL of the ginger extract was added with continuous stirring for 30 min. Gradual addition of 1 M NaOH resulted in the formation of a pale-yellow-colored precipitate. The precipitate was then centrifuged at 7500 rpm for 10 min, and the resulting white ZnO nanoparticles were collected. Subsequently, the nanoparticles were dried at 70 ℃ and calcined at 400 ℃ for 4 h [40].

Similarly, for α-Fe_2_O_3_ NPs, weight of 3 g of FeSO_4_·7H_2_O was dissolved in 300 mL of deionized water to achieve a final concentration of 0.1 M. Following the addition of 20 mL of ginger extract with continuous stirring for 30 min, 1 M NaOH was gradually added until the blue-colored solution turned grey. The resulting solution was centrifuged at 7500 rpm for 10 min, and the collected black-reddish α-Fe_2_O_3_ NPs were dried at 95 ℃ for 4 h. Finally, the NPs were calcined at 400 ℃ for 4 h [41].

The prepared NPs were characterized in our previous publications [38, 39] using atomic force spectroscopy, scanning electron microscopy, X-ray diffraction, zeta-potential analysis, Fourier-transform infrared spectroscopy, and UV–Visible spectroscopy [38, 39].

### Photoluminescence (PL) spectroscopy

To investigate the intrinsic and extrinsic transitions of the metal oxide nanoparticles, photoluminescence (PL) spectroscopy was employed. PL measurements were conducted using either a single-pass 0.5 m prism monochromator or a 0.32 m grating monochromator with a photomultiplier detector for visible and ultraviolet (UV) measurements. For the infrared (IR) spectrum, a thermoelectrically cooled InGaAs detector was utilized.

The PL measurements were performed at varying temperatures using a closed-cycle He-cooled cryostat equipped with quartz windows. Several UV excitation sources were employed, including a HeCd laser operating at 325 nm, a UV line from an Ar-ion laser at 351.1 nm, and a pulsed N_2_ laser with sub-nanosecond pulses operating at 337 nm. In the case of the Er-doped section, the IR excitation source was an InGaAs laser diode operating at 983 nm or a tunable Ti:sapphire laser. The laser spot sizes were smaller than 1 mm in diameter.

The PL signals were detected using the lock-in technique, which allows for the selective detection of the desired signals amidst background noise [42]. This technique enhances the sensitivity and accuracy of the measurements, enabling the precise characterization of the photoluminescent properties of the metal oxide nanoparticles.

### DFT and TD-DFT calculations studies

The DFT calculations for the ground-state geometry optimization of α-Fe_2_O_3_ and ZnO NPs were performed at the B3LYP level of theory [43]. The 6-311++G(d,2p) basis set was employed for these calculations, and the Gaussian 03 software package was utilized [44]. These calculations aimed to determine the most stable geometries of α-Fe_2_O_3_ and ZnO NPs based on their ground-state electronic structures. Additionally, molecular electrostatic potential (MEP) maps were generated for α-Fe_2_O_3_ and ZnO NPs using the same level of theory used for geometry optimization. MEP maps provide insights into the distribution of the electrostatic potential across the molecular surfaces and are useful for understanding the reactivity and chemical properties of the NPs.

To explore the frontier molecular orbitals (FMOs) and their corresponding energies, TD-DFT calculations were conducted [45]. These calculations were performed in accordance with Koopman's theorem [46], which allows for the determination of the vertical excitation energies corresponding to the transitions between molecular orbitals. The TD-DFT calculations provide valuable information about the electronic transitions and optical properties of α-Fe_2_O_3_ and ZnO NPs.

By employing these computational techniques, we aimed to gain a comprehensive understanding of the ground-state geometry, molecular electrostatic potential, and electronic transitions of α-Fe_2_O_3_ and ZnO NPs, contributing to the characterization of their structural and electronic properties.

### Metal oxides and human ferritin

This study was conducted at Ibn Al-Balady Hospital in Baghdad, between December 2021 and January 2022. A total of sixty diabetic volunteers with anemia (31 women and 29 men) aged 35–50 years were recruited for this study. Additionally, thirty healthy volunteers (15 females and 15 males) within the same age range were enrolled as controls. The control group consisted of individuals diagnosed by physicians as non-diabetic, without hypertension, not currently experiencing acute illness, and having no history of alcohol consumption or smoking.

To assess the impact NPs on serum ferritin levels, measurements were taken before and after the addition of a suspension of NPs with a molar ratio of 1:1 (serum to NPs oxide) in an in vitro setting. The serum ferritin levels were determined using the VIDAS Ferritin (FER) assay, following a standardized protocol [47]. By comparing the ferritin levels before and after the addition of metal oxide NPs, the study aimed to investigate any changes induced by the NPs in both the diabetic volunteers with anemia and the control group.

### Statistical analysis

Statistical analysis was performed using the unpaired Student t-test with *Welch's correction*, employing a two-tailed approach. This test was utilized to assess the statistical significance and describe the differences between the groups under investigation, considering a significance level of *p* < 0.05. Additionally, a Pearson correlation test was employed to explore potential correlations between the different groups. All statistical analyses were conducted using GraphPad Prism 9 software [48] for Mac, with the year 2021 version.

### Molecular docking

In silico assays were conducted as part of this study. The crystal structure of human H-chain ferritin (PDB code: 2FHA) was obtained from the Protein Data Bank [49]. Human H-chain ferritin was selected as the target protein due to its significance in relation to obesity and diabetes, as supported by previous studies [50].

To prepare the NPs and the target protein for the docking simulations, AutoDockTools-1.5.7 software was utilized [51]. The NPs and the target protein underwent preprocessing steps using this software. The target protein file was prepared by adding polar hydrogens and Kollman charges, and the resulting file was saved in pdbqt format, which is suitable for molecular docking simulations.

Molecular docking calculations were performed using AutoDockTools-1.5.7 software. The docking results and subsequent visualization studies were conducted using Discovery Studio 2021 software [52]. These tools enabled the analysis of the docking interactions between the NPs and the target protein, providing insights into their binding modes and potential inhibitory effects on human H-chain ferritin.

## Results and discussion

### Photoluminescence (PL) spectroscopy

**Fig. 1:**
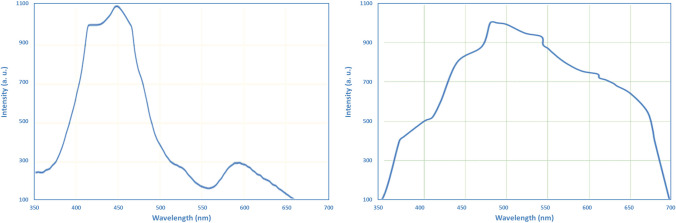
Photoluminescence (PL) spectra of ZnO NPs (left) and α-Fe_2_O_3_ NPs (right) prepared from ginger extract

The metal oxides, which were synthesized from ginger extract and characterized in previous studies [38, 39], were further analyzed using photoluminescence (PL) spectroscopy. Figure 1 presents the solid-state photoluminescence spectra, illustrating the emission energy gap of the metal oxide nanoparticles.

For ZnO NPs (Fig. 1a), two distinct emission peaks were observed in the visible region at 450 nm (E_g_ = 2.7 eV) and 590 nm. The emission peak in the visible region can be attributed to crystal defects, such as Zn-interstitials and oxygen vacancies (VO) [53]. Oxygen vacancies result from the loss of oxygen atoms from their respective positions in the crystal lattice, and they can exist in both the bulk and the surface or subsurface of nanomaterials. The presence of these defects contributes to the observed emission in the visible range. The energy gap (E_g_) of the ZnO NPs can be calculated using Eq. 1, where E_g_ represents the energy gap and x denotes the maximum wavelength [54].

1 $${\text{Eg}}\;({\text{eV}}) = \frac{1240}{x}$$

On the other hand, α-Fe_2_O_3_ NP exhibited a single emission peak in its PL spectrum (Fig. 1b). The peak appeared at 485 nm (E_g_ = 2.5 eV), indicating the photoluminescence emission of α-Fe_2_O_3_ at room temperature. This emission peak is associated with the successful formation of α-phase iron oxide, confirming the presence of the desired crystal structure [55].

These PL measurements provide valuable information about the photoluminescent properties of the metal oxide nanoparticles, indicating the presence of crystal defects and the successful formation of the desired crystal structure for ZnO and α-Fe_2_O_3_, respectively.

### DFT and TD-DFT calculations studies

**Fig. 2:**
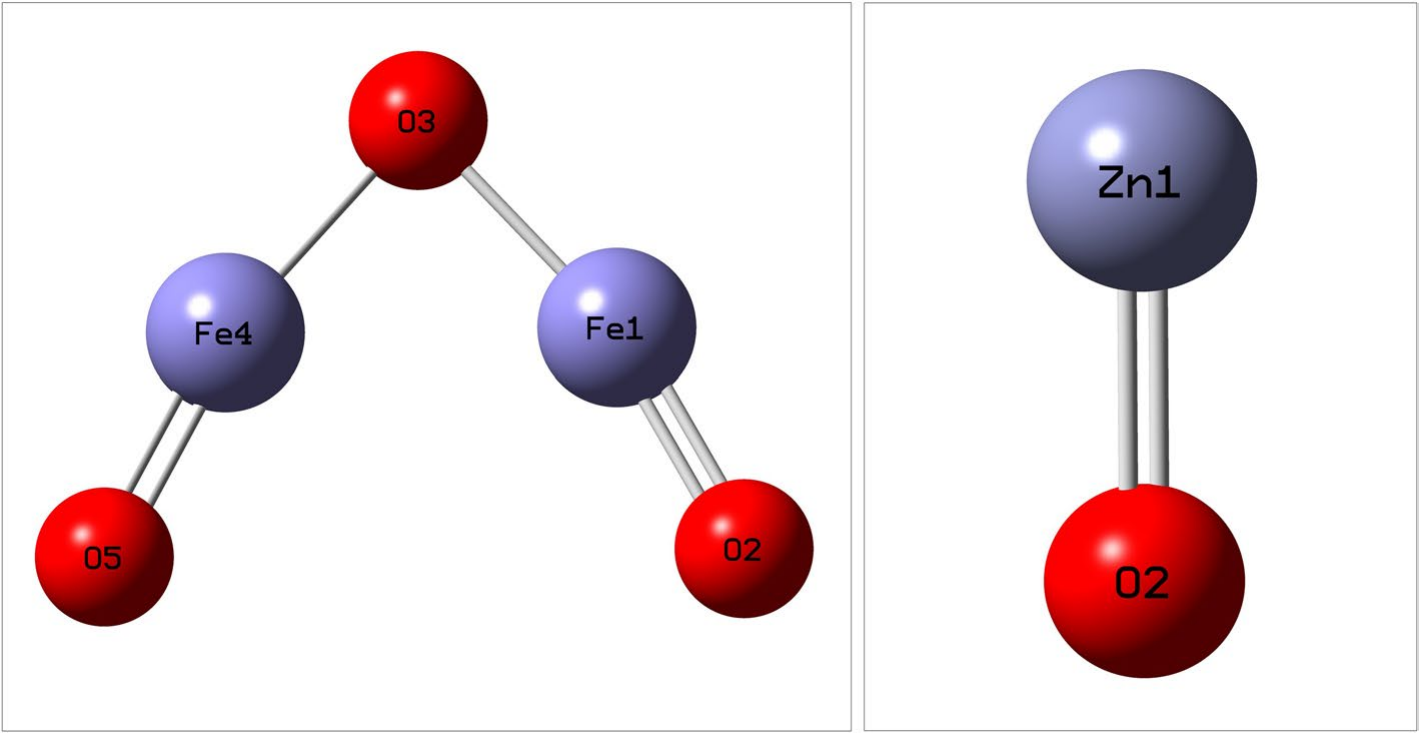
Optimized structures of α-Fe_2_O_3_ and ZnO nanoparticles

The fully optimized structures of α-Fe_2_O_3_ and ZnO nanoparticles, obtained through B3LYP/6-311++G(d,2p) level of theory calculations, are depicted in Fig. 2. For α-Fe_2_O_3_ NPs (Fig. 2a), the Fe–O single bond length is 1.73 Å, while the Fe=O double bond length is 1.54 Å. The Fe–O–Fe and O–Fe–O angles are found to be 85.1° and 166.2°, respectively. These values reflect the balanced electrostatic interactions between oppositely charged atoms within the Fe_2_O_3_ structure. Similarly, the bond length between the two atoms in ZnO nanoparticles (Fig. 2b) is 1.72 Å. The calculated bond lengths and angles are in agreement with the reported values [56, 57].

The dipole moments of α-Fe_2_O_3_ and ZnO were determined to be 2.65 and 5.80 Debye, respectively. This indicates that ZnO exhibits a stronger molecular interaction with various force fields compared to α-Fe_2_O_3_, as ZnO possesses a higher polarity.

**Fig. 3:**
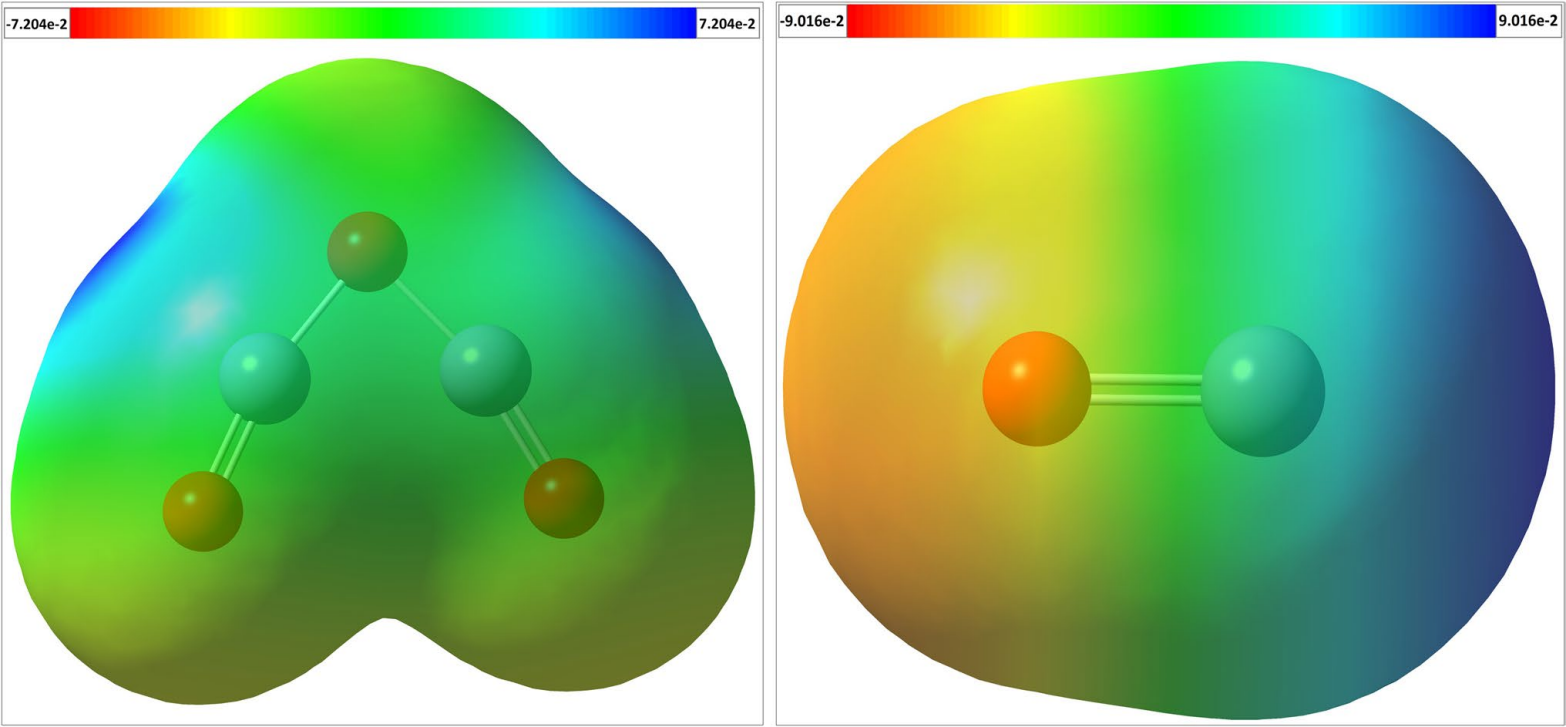
Molecular electrostatic potential maps for α-Fe_2_O_3_ and ZnO. The red color indicates regions with the most electronegative potential, while the blue color represents the most positive electrostatic potential regions. Zero-potential regions are denoted by the green color

Molecular electrostatic potential (MEP) maps were generated for both oxides to visualize the distribution of positive and negative potentials, identifying potential sites for nucleophilic and electrophilic attacks as well as hydrogen bonding interactions [58]. The MEP map of α-Fe_2_O_3_ (Fig. 3a) displays blue regions representing positive potentials associated with iron atoms, red regions denoting negative potentials located on terminal oxygen atoms (=O), and a green region indicating zero potential on the central oxygen atom (–O–). In the case of ZnO (Fig. 3b), positive potentials are observed over the oxygen atom, while negative potentials are localized on the zinc atom. The negative potential zones are nucleophilic regions, ideal for electrophilic interactions with positively charged species, whereas the positive potential regions offer electrophilic regions suitable for interactions with negatively charged nucleophiles. The unshared electron pair on the oxygen atom may be attacked nucleophilically within the region of zero potential [59, 60].

**Fig. 4:**
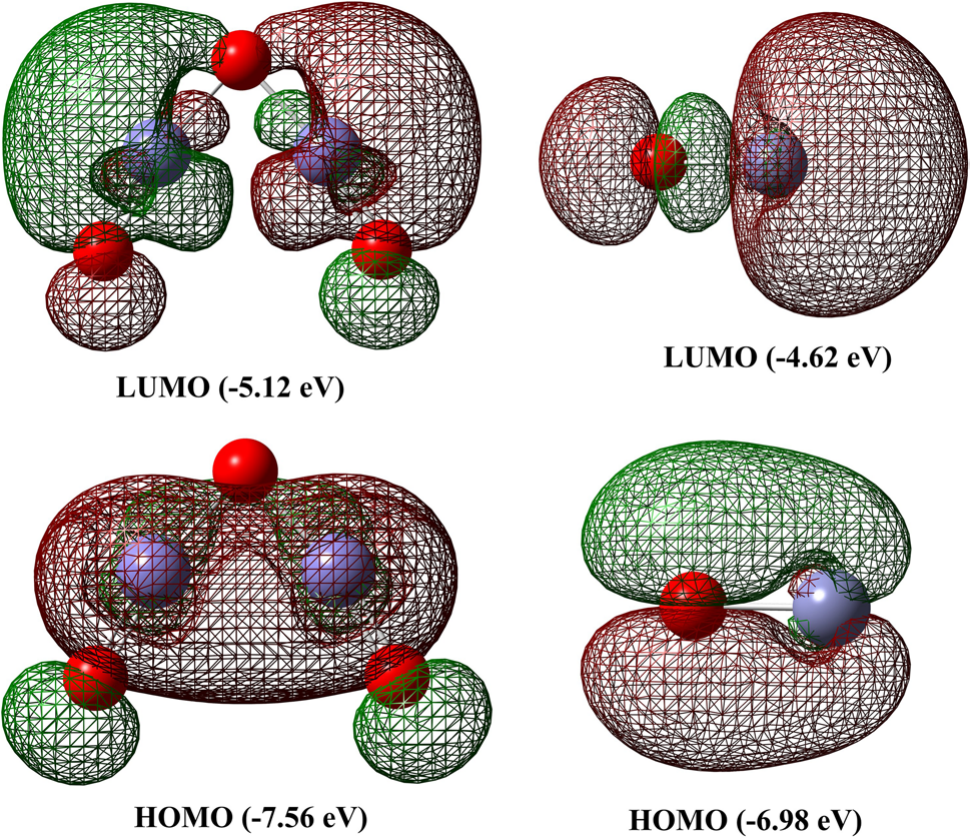
LUMO and HOMO molecular orbitals of α-Fe_2_O_3_ and ZnO

The frontier molecular orbitals (FMOs) of the two metal oxides were determined through TD-DFT calculations to assess their reactivity and biological activity. The highest occupied molecular orbital (HOMO) and the lowest unoccupied molecular orbital (LUMO) were examined (Fig. 4). These orbitals play a crucial role in determining the reactivity of compounds. Based on the LUMO and HOMO energy values, various parameters such as energy gap (ΔE_gap_), chemical potential (Π), electron affinity (A), hardness (η), absolute softness (σ), global softness (S), electrophilicity index (ω), Mulliken electronegativity (x), and ionization energy (Ip) were calculated according to Eqs. 2–10 and summarized in Table 1.

**Table 1:** Some quantum chemical parameters for Fe_2_O_3_ and ZnO

Parameter	α-Fe_2_O_3_	ZnO
E_LUMO_ (eV)	− 5.12	− 4.62
E_HOMO_ (eV)	− 7.56	− 6.98
∆E_gap_ (eV)	2.44	2.36
Electron affinity, A (eV)	5.12	4.62
Ionization energy, I_p_ (eV)	7.56	6.98
Hardness, η (eV)	1.22	1.18
Absolute softness, σ (eV)	0.82	0.85
Global softness, S (eV)	0.41	0.43
Mulliken electronegativity, X (eV)	6.34	5.80
Chemical potential, P_i_ (eV)	− 6.34	− 5.80
Electrophilicity index, ω	16.48	14.47
Total dipole moment, µ (D)	2.65	5.80

The LUMO is inclined to accept electrons, while the HOMO has a tendency to donate electrons. Therefore, their energy values are directly related to electron affinity (A) and ionization energy (Ip), respectively. ΔE_gap_ serves as a crucial determinant of the chemical reactivity of compounds, where a smaller value indicates lower stability and higher reactivity [48]. In this study, ZnO (ΔE_gap_ = 2.36 eV) was found to be more chemically reactive than α-Fe_2_O_3_ (ΔE_gap_ = 2.44 eV). The global softness (S), chemical hardness (η), and absolute softness (σ) values are important indicators of chemical stability. Higher hardness and lower softness values suggest greater stability of the system. Therefore, α-Fe_2_O_3_ (η = 1.22 eV, σ = 0.82 eV, and S = 0.41 eV) is considered more stable than ZnO (η = 1.18 eV, σ = 0.85 eV, and S = 0.43 eV). The higher values of electrophilicity index (ω) and Mulliken electronegativity (x) for α-Fe_2_O_3_ (x = 6.34 eV and ω = 16.48) compared to ZnO (x = 5.80 eV and ω = 14.47) support the notion that α-Fe_2_O_3_ exhibits lower biological activity than ZnO [30, 59].

2 $$\Delta {\text{E}}_{{{\text{gap}}}} = {\text{E}}_{{{\text{LUMO}}}} - {\text{E}}_{{{\text{HOMO}}}}$$

3 $${\text{A}} = - {\text{E}}_{{{\text{LUMO}}}}$$

4 $${\text{I}}_{{\text{p}}} = - {\text{E}}_{{{\text{HOMO}}}}$$

5 $${\upeta } = \frac{{\Delta {\text{E}}_{{{\text{gap}}}} }}{2}$$

6 $$\sigma = \frac{1}{{\upeta }}$$

7 $$S = \frac{1}{{2{\upeta }}}$$

8 $${\text{X}} = \frac{{ - \left( {{\text{E}}_{{{\text{HOMO}}}} + {\text{E}}_{{{\text{LUMO}}}} } \right)}}{2}$$

9 $${\text{P}}_{{\text{i}}} = - {\text{X}}$$

10 $${\upomega } = \frac{{{\text{P}}_{{\text{i}}}^{2} }}{{2{\upeta }}}$$

**Fig. 5:**
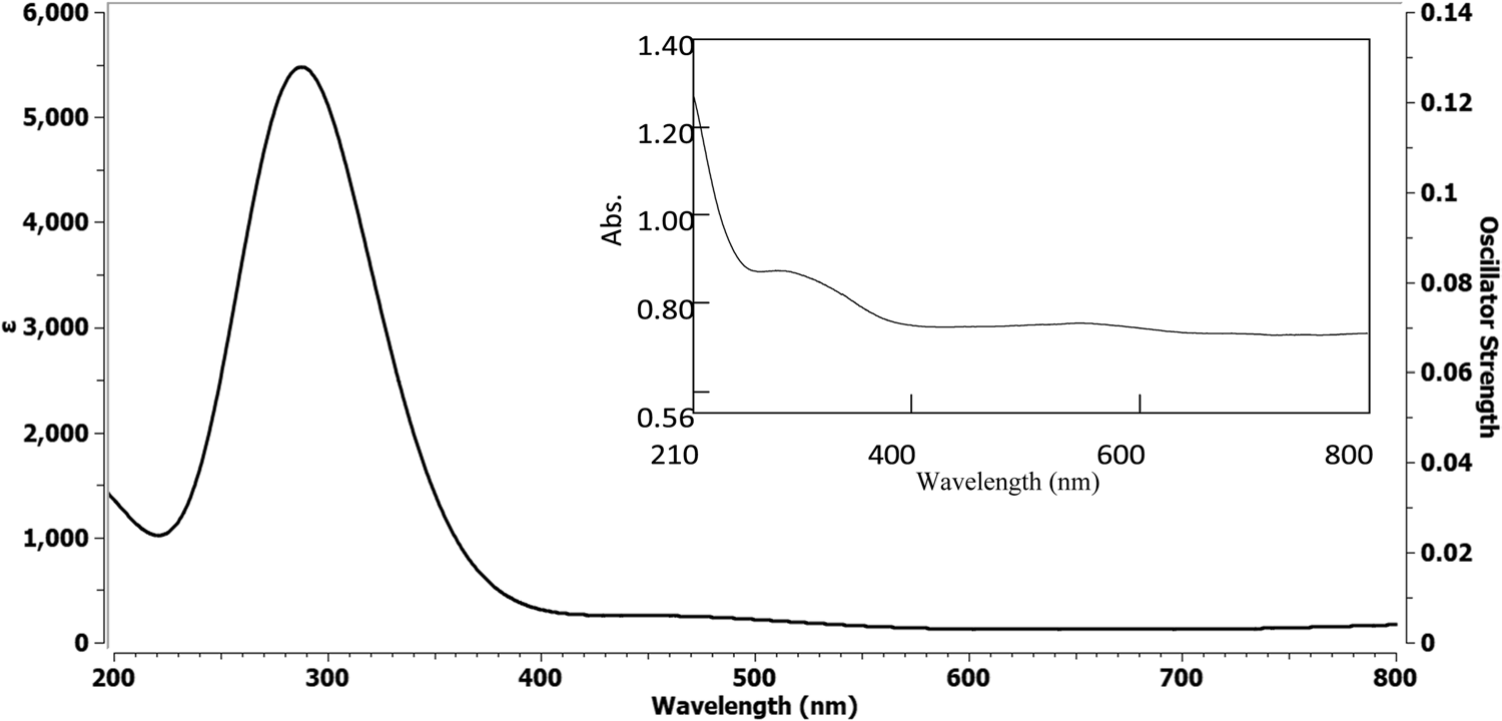
UV–VIS spectrum of α-Fe_2_O_3_ in water: calculated (left) and experimental (right)

**Fig. 6:**
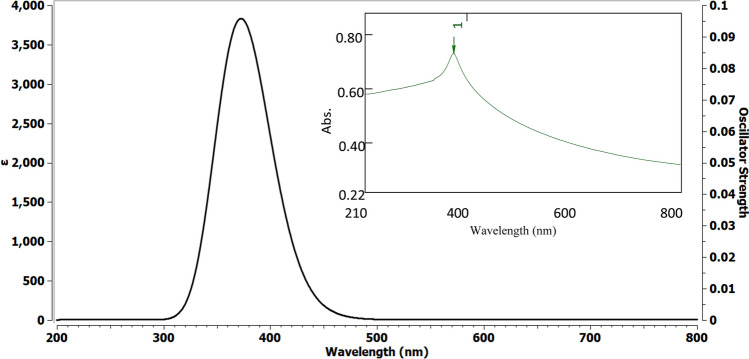
UV–VIS spectrum of ZnO in water: calculated (left) and experimental (right)

Theoretical electronic absorption spectra of the two oxides in water were calculated and compared to the experimentally obtained spectra. The calculated UV–Vis spectrum of α-Fe_2_O_3_ (Fig. 5) displayed a peak at 288 nm, in good agreement with the experimental spectrum that exhibited a peak at 282 nm. Similarly, the experimental UV–Vis spectrum of ZnO (Fig. 6) demonstrated a peak at 375 nm, which was strongly supported by the calculated spectrum showing a peak at 371 nm.

### Metal oxides NPs and the human ferritin

**Fig. 7:**
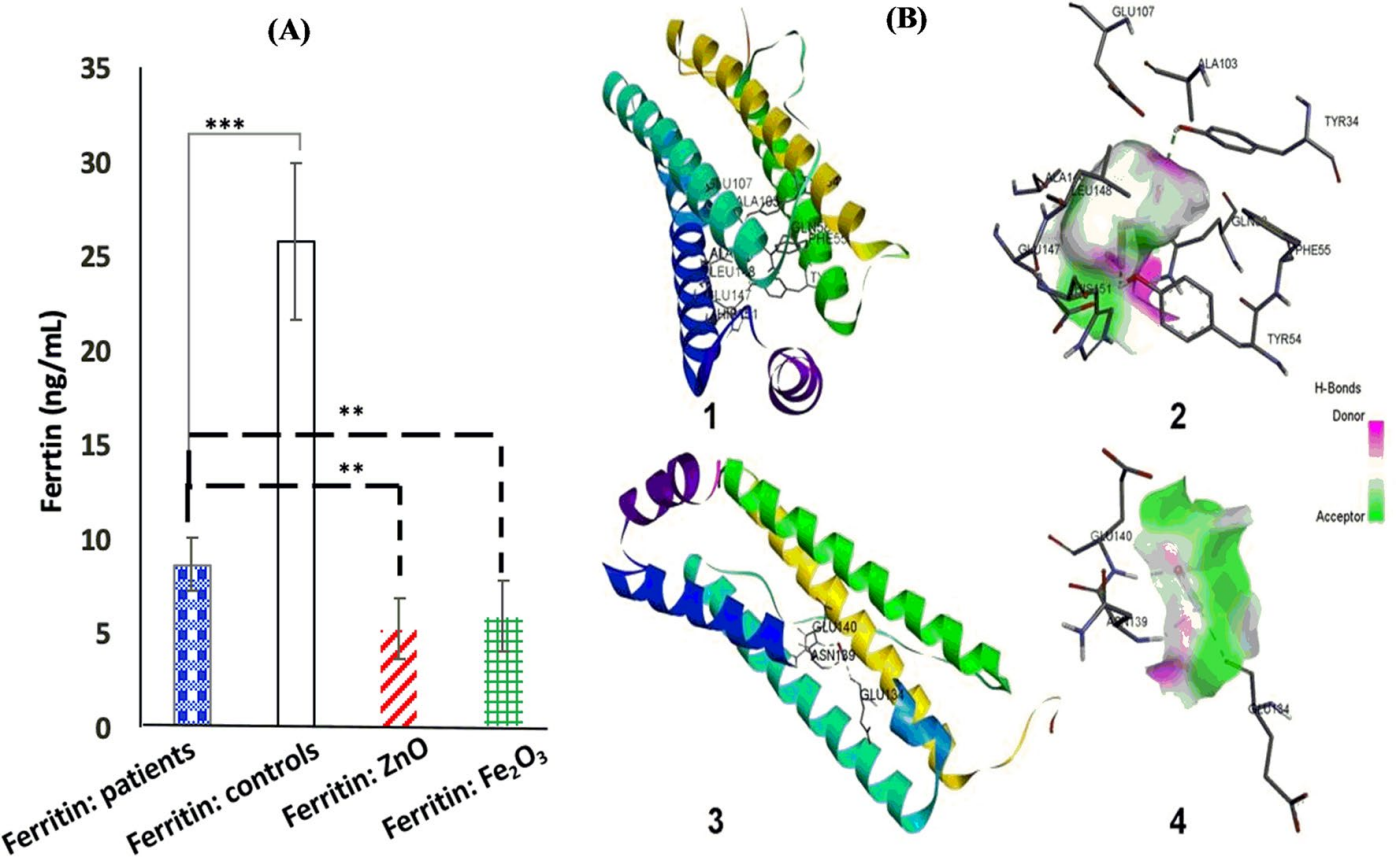
Effect of MO NPS and their interactions at the active site of human H-chain ferritin. **A** Ferritin levels in diabetic patients before and after in vitro addition of ZnO and Fe_2_O_3_, **B** molecular docking of (**1**) α-Fe_2_O_3_ NPs and (**3**) ZnO NPs and hydrogen bond interactions between NPs and the active site of human H chain ferritin (**2**: α-Fe_2_O_3_ NPs and **4**: ZnO NPs)

The initial ferritin levels in patients before the addition of metal oxide NPs were significantly lower compared to the control group (mean ± SD = 8.58 ± 1.39 ng/dL vs 25.73 ± 4.12 ng/dL, *p* < 0.001). After treatment with ZnO NPs and α-Fe_2_O_3_ NPs, the ferritin levels decreased significantly to 5.21 ± 1.57 ng/dL and 5.8 ± 1.8 ng/dL, respectively (*p* < 0.0001, Fig. 7A). This indicates that both ZnO NPs and α-Fe_2_O_3_ NPs have an inhibitory effect on serum ferritin levels, which is contrary to previous studies [61, 62].

The external surface of ferritins can be modified chemically and genetically, making it a potential site for targeting peptides. The treatment of serum ferritin with metal oxide NPs led to a partial loss of kinetic comparison compared to metal oxide NPs-free ferritin. This may be attributed to the proximity of bound metal ions at positions His136 and Lys68 in ferritin to the ferroxidase site, which can influence the catalytic reaction of ferritin. Additionally, since ferritin is a protein composed of polypeptide chains linked by amide bonds, interactions between these amide groups and the oxygen in metal oxides may affect the folding of ferritin and cause the unfolding of the polypeptide chains, resulting in reduced ferritin levels. Alternatively, there may be ionic or covalent bonds formed between metal oxides and functional groups and amino acid residues in the proteins.

The binding energy values between α-Fe_2_O_3_ and ZnO with human H-chain ferritin were calculated as − 3.2 kcal/mol and − 2.1 kcal/mol, respectively. This indicates that α-Fe_2_O_3_ has a higher binding affinity to human H-chain ferritin. α-Fe_2_O_3_ interacts through hydrogen bonding with TYR34 and TYR54 amino acid residues of human H-chain ferritin (Fig. 7B, 1 and 2, Table 2), with bond lengths of 2.55 Å and 2.18 Å, respectively. On the other hand, ZnO forms a hydrogen bond with the Glu140 amino acid residue of human H-chain ferritin (Fig. 7B, 3 and 4, Table 2), with a bond distance of 1.93 Å. Molecular docking studies allow for the detection of hydrogen bonds, electrostatic, and hydrophobic interactions between a ligand and a target. In this case, α-Fe_2_O_3_ and ZnO were found to interact with human H-chain ferritin only through hydrogen bonds, and the distances of these hydrogen bonds indicate strong interactions. These MO NPs (ZnO NPs and Fe_2_O_3_) have interestingly chose in this study due to their wide spectra of biological application because of their exceptional biocompatibility, good economic, and diminished toxicity effect. They also showed excellent biomedical applications, such as anticancer, antibacterial, drug delivery, antidiabetic effect, anti-inflammation and wound healing [63–67].

**Table 2:** The docking scores and the interacting residues of 2FHA proteins with the α-Fe_2_O_3_ and ZnO

Target protein	Compound	Interaction type	Interacting amino acid	Bond distance	Binding energy (kcal/mol)
2FHA	α-Fe_2_O_3_	Conventional hydrogen bond	Tyr34	2.54 Å	− 3.2
			Tyr54	2.18 Å	
	ZnO	Conventional hydrogen bond	Glu140	1.93 Å	− 2.1
		Metal-acceptor	Glu134		

## Conclusion

In conclusion, this study utilized DFT calculations and molecular docking simulations to investigate the properties and interactions of ZnO and α-Fe_2_O_3_ nanoparticles with human H-chain ferritin. The DFT calculations provided insights into the optimized structures, dipole moments, and molecular electrostatic potential maps of the two oxides. ZnO exhibited a higher dipole moment, indicating stronger molecular interactions with various force fields compared to α-Fe_2_O_3_. The MEP maps revealed regions of positive and negative potentials, allowing for nucleophilic and electrophilic interactions with different charged species. TD-DFT calculations provided information about the frontier molecular orbitals and quantum chemical parameters of the oxides. The results indicated that ZnO has higher chemical reactivity and biological activity compared to α-Fe_2_O_3_. Molecular docking simulations further confirmed the interaction between the metal oxide ligands and the human H-chain ferritin target, primarily through hydrogen bonding interactions. Considering the combined in silico and experimental results, ZnO emerges as the suggested biological target for ferritin. The findings from this study contribute to the understanding of the properties and interactions of metal oxide nanoparticles with human ferritin and can inform future research in the field of nanomedicine, however, further studies on their toxicity are required.

## Data Availability

All data generated or analyzed during this study are included in this published article.
